# Synthetic TGF-β Signaling Agonist-Treated Dendritic Cells Induce Tolerogenicity and Antirheumatic Effects

**DOI:** 10.3390/cimb44090261

**Published:** 2022-08-24

**Authors:** Ji-Soo Oh, Sung-Uk Hwang, Kyung-Eun Noh, Jun-Ho Lee, So-Yeon Choi, Ji-Hee Nam, Min-Seon Song, Nam-Chul Jung, Jie-Young Song, Han Geuk Seo, Younghwa Na, Dae-Seog Lim

**Affiliations:** 1 Department of Biotechnology, CHA University, 335 Pangyo-ro, Bundang-gu, Seongnam 13488, Korea; 2 Pharos Vaccine Inc., 14 Galmachiro 288 bun-gil, Jungwon-gu, Seongnam 13201, Korea; 3 Department of Radiation Cancer Sciences, Korea Institute of Radiological and Medical Sciences, 75 Nowon-ro, Nowon-gu, Seoul 01812, Korea; 4 Department of Food Science and Biotechnology of Animal Products, Sanghuh College of Life Sciences, Konkuk University, 120 Neungdong-ro, Gwangjin-gu, Seoul 05029, Korea; 5 College of Pharmacy, CHA University, 120 Haeryong-ro, Pocheon 11160, Korea

**Keywords:** TGF-β signaling, dendritic cells, regulatory T cells, autoimmune diseases, rheumatoid arthritis

## Abstract

The newly synthesized compound TGF-β signaling agonist (T74) is a small molecule associated with the TGF-β receptor signaling pathway. Tolerogenic dendritic cells (tDCs) have been used to examine immunosuppressive and anti-inflammatory effects in multiple autoimmune disease models. The aim of this study was to investigate whether treatment of DCs with T74 has an antirheumatic effect in a mouse model of collagen-induced arthritis (CIA). Bone marrow-derived cells were obtained from DBA/1J mice and differentiated into DCs. T74-treated DCs (T74-DCs) were generated by treating bone marrow-derived DCs with LPS, type II collagen, and T74. T74-DCs expressed lower levels of surface molecules and inflammatory cytokines associated with antigen presentation and T cell stimulation. The ability of T74-DCs to differentiate effector T cells was lower than that of T74-untreated DCs (NT-DCs), but T74-DCs increased the regulatory T (Treg) cell differentiation in vitro. DBA/1J mice received two subcutaneous (s.c.) injections of type II collagen to establish CIA. Mice then received two s.c. injections of T74-DCs or NT-DCs. Joint inflammation was ameliorated in the paws of T74-DC-treated mice. Additionally, Treg populations in T74-DC-treated mice were higher than in NT-DC-treated or PBS-treated CIA mice. Taken together, these results demonstrate that T74 induces tolerance in DCs, and that T74-mediated DCs exert antirheumatic effects via induction of Tregs.

## 1. Introduction

TGF-β receptor signaling molecules have been synthesized for the treatment of cancers and fibrosis [1,2,3]. The small molecule inhibitor of TGF-β receptor I (TGFBRI) called galunisertib showed higher disease control in phase Ib/IIa when combined with radiochemotherapy in malignant glioma treatment [4]. On the other hand, some studies have examined other TGF-β receptor agonists as a treatment for autoimmune disease. *H. polygyrus*-derived TGF-β receptor agonist induces expansion of murine Foxp3^+^ regulatory T (Treg) cells in vitro and in vivo, and activin A (another TGF-β superfamily member) increases phagocytosis of primary murine microglial cells [5,6]. The TGF-β signaling agonist (T74, (E)-1-(3-Aminophenyl)-3-(2,5-dimethoxyphenyl)prop-2-en-1-one) used in this study has been newly synthesized [7,8] and consequently surmised to induce tolerogenicity of antigen-presenting cells (APCs) in the immune system in acting as an agonist of TGF-β signaling.

Dendritic cells (DCs) are the most professional APCs and are essential orchestrators of immunological homeostasis. Immature DCs (imDCs) in the periphery recognize antigens and present them to T or B lymphocytes to stimulate proliferation, differentiation, and activation. DCs comprise heterogeneous subsets that play different roles in immune responses depending on their maturation status. Fully mature DCs (mDCs) conduct immunofacilitatory roles that promote immunogenic effector T cells, whereas tolerogenic DCs (tDCs) have immunosuppressive roles that regulate overactivated immune responses by differentiating systemic Tregs [9]. Prior studies show that TGF-β signaling induces tolerogenic properties in DCs. TGF-β receptor II (TGFBRII)-deficient DCs are pro-inflammatory, activate lymphocytes, and inhibit differentiation of antigen (Ag)-specific Tregs, leading to spontaneous multiorgan autoimmunity [10]. Imbalances of immune homeostasis mediated by TGF-β signaling-deficient DCs were demonstrated in mouse models of experimental autoimmune encephalomyelitis and autoimmune pancreatitis [11,12]. Because tDCs have been used to examine immunosuppressive and anti-inflammatory properties in multiple autoimmune disease models [9,13], we selected DCs as a means of identifying the immunosuppressive properties of T74.

Here, we evaluated the tolerogenicity of T74-treated DCs (T74-DCs) and confirmed the therapeutic effects of T74-DC therapy in a collagen-induced arthritis (CIA) model.

## 2. Materials and Methods

### 2.1. Mice and Ethics Approval

The Institutional Animal Care and Use Committee (IACUC) of CHA University approved the protocols for the animals in this study (Project No. IACUC200149), and all animal experimentations were conducted according to the approved protocols. Female DBA/1J mice (6–8 weeks old) were purchased from Orient Bio, Inc. (Seongnam, Korea). Mice (3–5 mice/cage) were managed in controlled environmental conditions (light/dark cycle, room temperature, and humidity).

### 2.2. TGF-β Signaling Agonist, T74

T74 ((E)-1-(3-Aminophenyl)-3-(2,5-dimethoxyphenyl)prop-2-en-1-one) was synthetized by Dr. Younghwa Na’s lab, CHA University (Figure S1) as previously described in the patent literature [7,8]. T74 was stored at room temperature and had low aqueous solubility. Stock solution was diluted in DMSO and then further diluted in Roswell Park Memorial Institute Medium (RPMI)-1640 culture media.

### 2.3. DC Generation Using T74

Bone marrow (BM)-derived cells were obtained from bones of hind limb of DBA/1J mice to generate DCs, as described in [14]. BM progenitors were cultured in RPMI-1640 containing HEPES (Cytiva, Hyclone Laboratories, Logan, UT, USA), 10% FBS, 55 nM 2-mercaptoethanol, antibiotics–antimycotics (Gibco, Life Technologies, Grand Island, NY, USA), 20 ng/mL recombinant (rm) GM-CSF, and rm interleukin (IL)-4 (JW CreaGene, Seongnam, Korea). Cells were cultured at 37 °C under 5% CO_2_. An equal amount of the same fresh medium was added on Day 3, and half of the culture medium was replaced on Day 6 with the fresh medium. To generate T74-treated DCs (T74-DCs), bone marrow cells were cultured in the above-mentioned medium and treated with 10 μM T74 from Day 3 of culture. At Day 8 of culture, T74-DCs were generated by additional incubation for 4 h with 1 μg/mL LPS, 50 μg/mL type II collagen (CII, Sigma-Aldrich, St. Louis, MO, USA), and 10 μM T74. T74-untreated DCs (NT-DCs) were cultured using the same method, but treated with the same volume of vehicle (DMSO) instead of T74. imDCs were cultured in the absence of T74, LPS, and antigens. After 8 days of culture, imDCs, NT-DCs, and T74-DCs were harvested and used for further studies.

### 2.4. Flow Cytometry

For phenotypic analysis, the surface molecules of DCs were stained with monoclonal antibody-conjugated fluorescent agent. Cells were incubated with the following antibodies at 4 °C for 20 min: anti-CD11c (clone N418, allophycocyanin (APC) or phycoerythrin (PE)), anti-CD80 (clone 16-10A1, PE), anti-CD86 (clone PO3, fluorescein-5-isothiocyanate (FITC)), and anti-MHC II (clone 2G9, PE) (BioLegend or eBioscience, San Diego, CA, USA). To examine T cell subpopulations, cells were stained with APC-conjugated anti-CD4 (clone RM4-5), and then cells were fixed and permeabilized using an Intracellular Staining Kit (BD Biosciences, San Diego, CA, USA or Invitrogen, Waltham, MA, USA). For intracellular staining, anti-IFN-γ (clone XMG1.2, PE), anti-IL-4 (clone 11B11, Alexa Fluor 488), or anti-IL-17A (clone TC11-18H10.1, PE) (all from BD Bioscience) plus anti-Foxp3 (clone FJK-16s; Invitrogen, PE) antibodies were used. FlowJo v.10.6.2 software (FlowJo, Ashland, OR, USA) was used to analyze the data.

### 2.5. Cytokine Measurement

Commercially available ELISA kits were used to confirm the cytokine secretion of dendritic cells and T cells. The following products were used for measurement: Mouse OptEIA ^TM^ IL-12p40, IL-10, IFN-γ (BD Biosciences), Mouse IL-1β, IL-4, IL-17A, TNF-α ELISA MAX ^TM^ deluxe (BioLegend), and Mouse TGF-β1 and IL-6 duoset ELISA (R&D Systems, Minneapolis, MN, USA).

### 2.6. Western Blot

Whole-cell lysates were prepared for Western blotting. Protein concentrations were measured using a Pierce™ Coomassie Plus (Bradford) Assay Reagent (Thermo Fisher Scientific, Waltham, MA, USA). The proteins were separated on polyacrylamide gel by SDS-PAGE and then transferred to PVDF membranes (Thermo Fisher Scientific). Then, 10% (*w*/*v*) skim milk in PBST was used for blocking the membranes and the membranes incubated overnight at 4 °C with the following specific primary antibodies (all diluted 1:1000): phosphorylated (p)ERK1/2, ERK1/2, p-JNK, JNK, p-p38, p38, p-NF-kB p65, and NF-kB p65 (Cell Signaling Technology, Danvers, MA, USA), p-TGFBRI, and TGFBRI (Thermo Fisher Scientific), and p-Smad2, Smad2 (Cell Signaling Technology), or GAPDH (Bioss, Woburn, MA, USA). HRP-conjugated anti-mouse or anti-rabbit secondary antibodies (diluted 1:5000; Santa Cruz Biotechnology, Dallas, TX, USA) were used for 2 h at room temperature. The signals were detected using the SuperSignal Chemiluminescence Substrate (Thermo Fisher Scientific) and c280 Imaging System (Azure biosystems, Dublin, CA, USA).

### 2.7. Reverse Transcriptase PCR and Real-Time PCR

Total RNA was extracted and used to synthesize cDNA using Labozol (Thermo Fisher Scientific) and the LaboPass cDNA synthesis kit (Cosmogenetech, Seoul, South Korea). The cDNA samples were analyzed by reverse transcriptase PCR (RT-PCR) and quantitative real-time PCR (qRT-PCR). SensiFast SYBR No-Rox Kit (Bioline, London, UK) was used for qRT-PCR. The following primers were used for PCR: *Ido* forward, CCT GCC TCC TAT TCT GTC TTA TG and *Ido* reverse, AGA ATG TCC ATG TTC TCG TAT GT; *Pdlim4* forward, TTG TCA AAG CGA GAG ACA AG and *Pdlim4* reverse, GTA CAG CCG TTC ATC TAG GA; *Rsad2* forward, GGT GCC TGA ATC TAA CCA GAA G and *Rsad2* reverse, CCA CGC CAA CAT CCA GAA TA; *Gapdh* forward, AAC AGC AAC TCC CAC TCT TC and *Gapdh* reverse, CCT GTT GCT GTA GCC GYA TT. Expression of *Ido* (a tDC function-related gene), *Pdlim4*, and *Rsad2* (specific markers of mDCs) mRNA was calculated as the relative quantity of the target mRNA divided by the relative quantity of *Gapdh* mRNA.

### 2.8. Co-Culture of DCs and Splenic T Cells

Isolated spleen from DBA/1J mice was disaggregated in RPMI-1640 medium. Splenocytes were filtered with a 70 µm cell strainer (BD Biosciences) and then the red blood cells were lysed using ACK Lysing Buffer (Lonza, Basel, Switzerland). T cells were enriched by passing the splenocytes through a nylon wool (Polysciences, Inc., Warrington, PA, USA) column. DCs and T cells were cultured directly in the same plates. Enriched T cells (1 × 10^6^ cells/mL) were employed as responders, and T74-DCs or NT-DCs (1 × 10^5^ cells/mL) were stimulators. T Cells and DCs were incubated at 37 °C for 72 h in RPMI-1640 medium containing 10% FBS.

### 2.9. CFSE Cell Proliferation Assay

Enriched T cells were stained with carboxyfluorescein succinimidyl ester (CFSE) using CFSE labeling kits (Molecular Probes, Eugene, OR, USA) according to the manufacturer’s instructions. CFSE-labeled cells were co-cultured with DCs as previously described. After 72 h, CFSE-labeled cells were detected in an FITC channel.

### 2.10. CIA Mouse Model

CIA was induced in DBA/1J mice as previously described [13]. The collagen emulsion was prepared by emulsifying chicken CII (Sigma-Aldrich) dissolved in 0.05 M acetic acid and complete Freund’s adjuvant (CFA, Sigma-Aldrich) at a 1:1 ratio. For initial immunization, 200 μg CII emulsified in CFA was injected s.c. in the skin of the tail. After 21 days, the booster was received in the same amount of CII emulsified in incomplete Freund’s adjuvant (Sigma-Aldrich). Then, the mice were s.c. administered 2 × 10^5^ CII-primed T74-DCs, NT-DCs, or PBS on Days 21 and 29. Arthritis incidence and footpad thickness were evaluated three times per week (every other day or third day) until Day 50 under double-blind conditions. The severity of arthritis was expressed as the mean arthritis index, scored as follows: 0 = normal; 1 = mild swelling of one or two toes; 2 = moderate swelling of several toes or the wrist or ankle; 3 = inflammation and edema involving entire paw; and 4 = maximal erythema and edema of the entire paw and deformation of joint leading to impaired function. Each mouse could receive a maximum score of 16 (sum of the score of all 4 paws). On Day 35, half of the mice from each group were selected randomly and sacrificed. The paws, inguinal lymph nodes, and spleens were isolated.

### 2.11. Histopathology

Mouse paws were fixed for 48 h in 4% paraformaldehyde. Fixed samples were processed sequentially as follows: decalcification for 21 days in an aqueous solution of hydrochloric acid (Sigma-Aldrich), dehydration in ethanol, clearing in Histoclear solution (National Diagnostics, Atlanta, GA, USA), infiltration, and embedding in paraffin. Serial sections (5 μm) were cut and stained with H&E. Histopathological features were examined under a Leica MC120 HD microscope camera (Wetzlar, Germany).

### 2.12. Statistical Analysis

Statistical significance was analyzed by Student’s *t*-tests or by one-way ANOVA followed by the Newman–Keuls test (GraphPad software; GraphPad Prism v.7.0, San Diego, CA, USA). Data are expressed as the mean ± SEM. A value of *p* < 0.05 was considered significant.

## 3. Results

### 3.1. Effect of TGF-β Receptor Signaling Agonist T74 on DC Maturation

**Figure 1:**
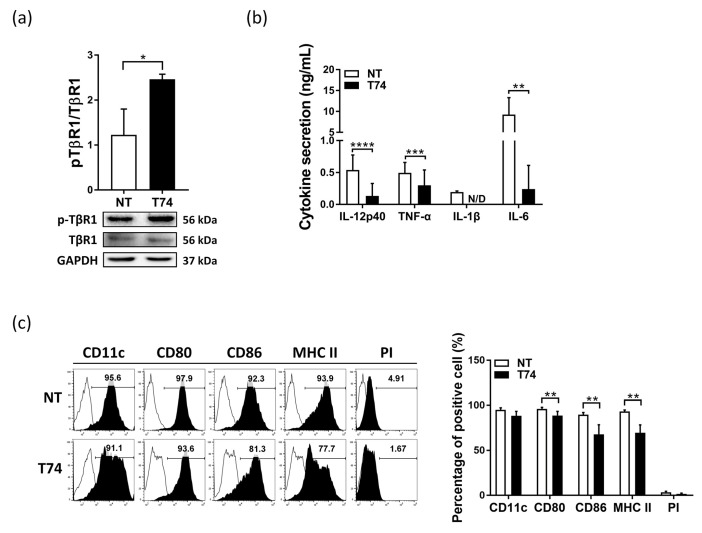
TGF-β receptor signaling agonist T74 blocks DC maturation. (**a**) Whole cell lysates pre-pared from T74-untreated DCs (NT) and T74-treated DCs (T74) were evaluated by western blotting for pTGFBRI (TβR1), TGFBRI. Values were normalized to GAPDH expression, n = 3. Data are representative of three independent experiments. (**b**) Pro-inflammatory cytokine secretion by T74-treated DCs (T74-DCs) was analyzed by ELISA, n = 5. (**c**) DCs were stained with fluorescence-conjugated antibodies specific for the indicated molecules and analyzed by flow cytometry. The histograms are representative of five independent mice. The bar graph shows the percentage of cells positive for each marker, n = 5. Data are expressed as the mean ± SEM. * *p* < 0.05, ** *p* < 0.01, *** *p* < 0.001, **** *p* < 0.0001. N/D: not detected.

Prior studies reported that tolerogenic DCs show a semi-mature phenotype [9]. To investigate the tolerogenicity of T74, we examined the effects of T74 on the maturation of DCs. First, the treatment conditions were established by treating DCs with T74 in five different doses (Figure S2). When T74 was treated with a dose of 50 μM or more in DCs, the cell viability decreased below 100% due to cytotoxicity. In addition, the pro-inflammatory cytokine (IL-12p40) production was significantly lower in the DC groups treated with T74 at a concentration of 10 μM or 25 μM compared to the NT-DC group. On the other hand, there was no significant difference in IL-12p40 production between the 10 μM group and the 25 μM group. Based on these results, we determined the T74 treatment concentration as 10 μM and applied it to subsequent experiments. As a previous study reported that early exposure of DCs to TGF-β contributes to greater suppressive effects on T cell activation compared with treatment at a later stage [15], we administered T74 to DCs from Day 3 of culture. To confirm that T74 acts as a TGF-β signaling agonist, we examined phosphorylation of TGFBRI in DCs by Western blotting. Phosphorylation of TGFBRI was higher in T74-DCs than in T74-untreated DCs (NT-DCs) (Figure 1a). Next, cytokine production and immunophenotypic analysis were performed to evaluate DC maturity. T74-DCs secreted significantly lower levels of pro-inflammatory cytokines (IL-12p40, TNF-α, IL-1β, and IL-6) than NT-DCs (Figure 1b). In addition, T74-DCs expressed significantly lower levels of CD80, CD86, and MHC class II than NT-DCs, without evidence of cell death or differentiation (as determined by PI and CD11c staining, respectively) (Figure 1c). These results indicate that T74 acts as a TGF-β receptor signaling agonist and blocks maturation of DCs.

### 3.2. Effect of T74-Treated DCs on T Cell-Mediated Immune Responses

**Figure 2:**
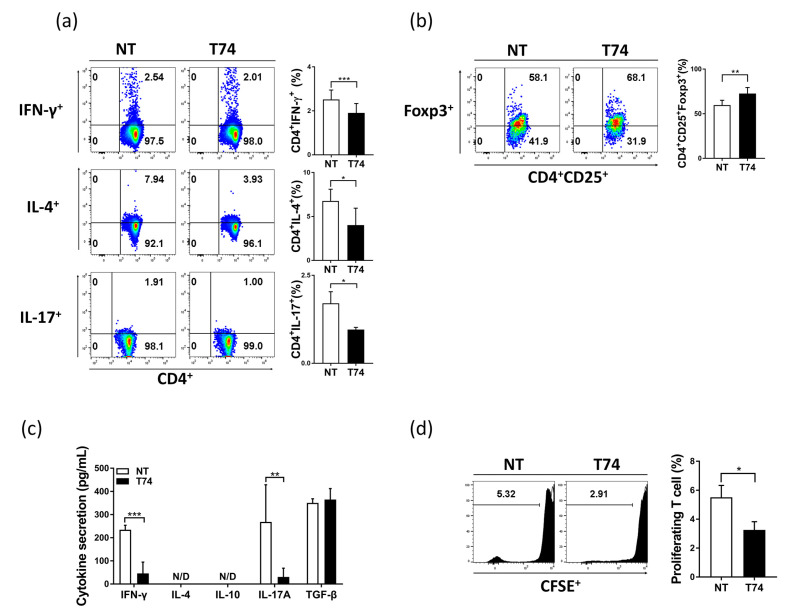
Immunosuppressive characteristics of T74-DCs. Enriched T cells were co-cultured with each DC subset at a ratio of 1:10 for 72 h. (**a**) T cell subpopulations were analyzed by flow cytometry. The percentage of induced Th1 (CD4^+^IFN-γ^+^), Th2 (CD4^+^IL-4^+^), Th17 (CD4^+^IL-17A^+^), and (**b**) Treg (CD4^+^CD25^+^Foxp3^+^) is shown on the dot plots. (**c**) Th1/Th2/Th17- and Treg-related cytokine levels in supernatants from DC/T cell co-cultures were measured by ELISA. (**d**) CD3^+^ T cells were stained with CFSE to measure T cell proliferation. All data are representative of four independent experiments and are expressed as the mean ± SEM, n = 4. * *p* < 0.05, ** *p* < 0.01, *** *p* < 0.001. N/D: not detected.

Next, we conducted a series of DC–T cell co-culture experiments to evaluate the effect of T74 on the function of DCs involved in T cell proliferation and polarization. Naïve CD3^+^ T cells were co-cultured with each DC subset for 72 h, and T cell subpopulations were examined by flow cytometry. T74-DCs reduced type 1 helper T cell (Th1), Th2, and Th17 populations significantly, but increased Treg populations markedly, compared with NT-DCs (Figure 2a,b). T cells cultured with T74-DCs (T74-DC-Ts) showed significantly lower secretion of Th1/Th17-mediated IFN-γ and IL-17A than T cells cultured with NT-DCs (NT-DC-Ts), but there was no significant effect on Treg-mediated TGF-β secretion (Figure 2c). Additionally, T74-DC-Ts were less proliferated than NT-DC-Ts (Figure 2d). These results show that T74 induces tolerogenic function of DCs.

### 3.3. Change in Expression of mDC-Specific Gene Markers by T74-DCs

**Figure 3:**
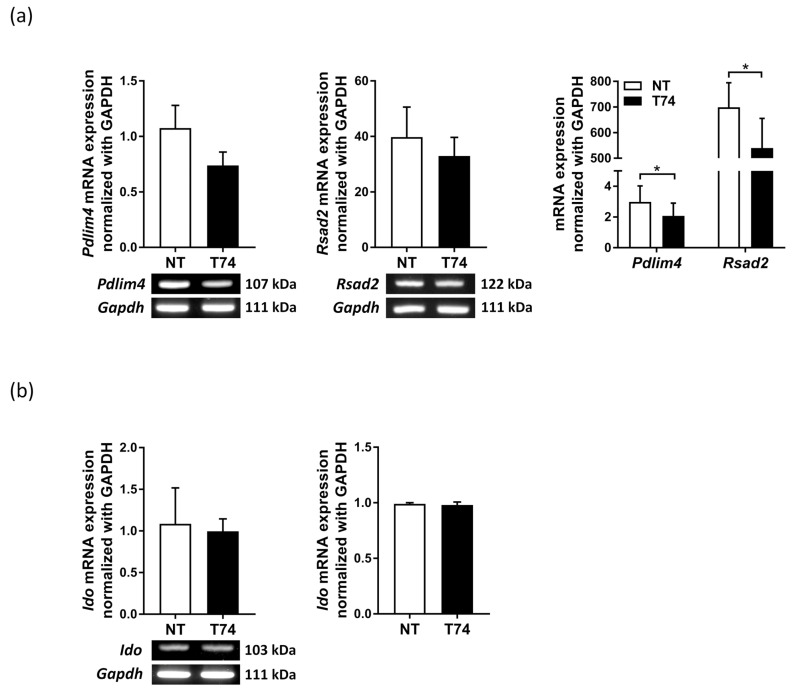
mDC markers *Pdlim4* and *Rsad2* were downregulated in T74-DCs. (**a**) Detection of *Pdlim4*, *Rsad2*, and (**b**) *Ido1* mRNA by RT-PCR (left) and qRT-PCR (right). Values in panels were normalized to *Gapdh* expression, n = 3. Data are representative of three independent DC preparations and are expressed as the mean ± SEM. * *p* < 0.05.

For further DC maturity assessment, we conducted RT-PCR to examine expression of *Pdlim4, Rsad2*, and *Ido* mRNA by T74-DCs. Compared with NT-DCs, T74-DCs expressed significantly lower levels of *Pdlim4* and *Rsad2* (mDC-specific gene markers) mRNA (Figure 3a). In the same context, qRT-PCR showed that levels of mRNA encoding *Pdlim4* and *Rsad2* in T74-DCs were significantly lower than those in NT-DCs (Figure 3a). However, there was no significant difference in *Ido* mRNA expression (Figure 3b).

### 3.4. DC Signaling Pathways Affected by T74

**Figure 4:**
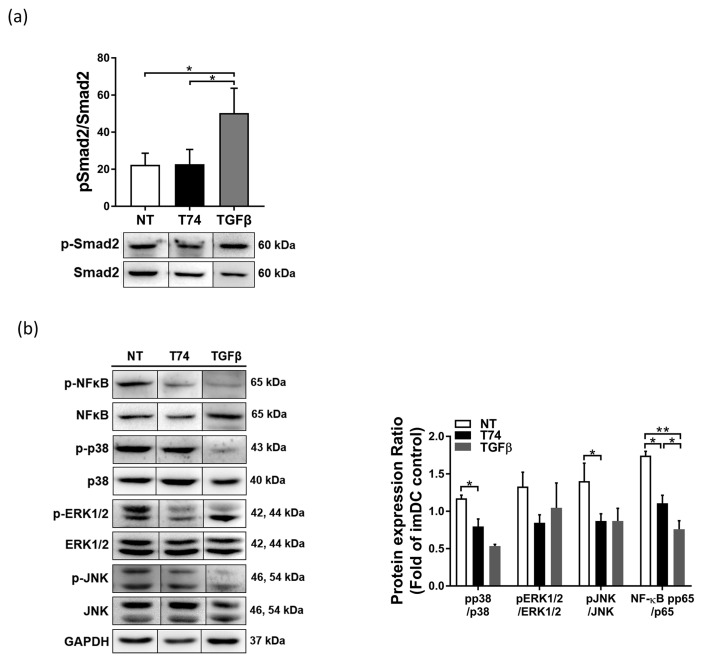
T74 suppresses MAPK and NFκB signal pathways. (**a**) Western blot analysis of pSmad2, Smad2, and GAPDH. Whole cell lysates prepared from T74-untreated DCs (NT), T74-DCs (T74), and recombinant (rm)TGF-β treated DCs (TGF-β). (**b**) Western blot analysis of NF-κBp65, p38, ERK1/2, and JNK. Phospho-protein expression was normalized to total expression of each respective protein. Fragments of the same image were spliced together to remove irrelevant lanes. Data in panels are representative of three independent experiments. The bar graphs indicate -fold expression (vs. imDCs), presented as the mean ± SEM, n = 3. * *p* < 0.05, ** *p* < 0.01.

Next, we investigated whether the canonical TGF-β/Smad signal pathway was affected by T74. Western blot analysis was performed to examine phosphorylation of Smad2. Although rmTGF-β-treated DCs showed markedly higher levels of Smad2 phosphorylation, T74-DCs showed levels similar to those in NT-DCs (Figure 4a). We then investigated how T74 regulates maturation of DCs by identifying signaling proteins involved in DC activation (NF-κB, p38, ERK1/2, and JNK MAPKs). Compared with NT-DCs, T74-DCs exhibited distinct defects in expression of phosphorylated NF-κB, p38, ERK1/2, and JNK (Figure 4b). These data imply that T74 affects phosphorylation of the MAPK and NF-κB pathways to a greater extent than Smad signaling.

### 3.5. Antirheumatic Effects of Type II Collagen-Primed T74-DCs on CIA Model

**Figure 5:**
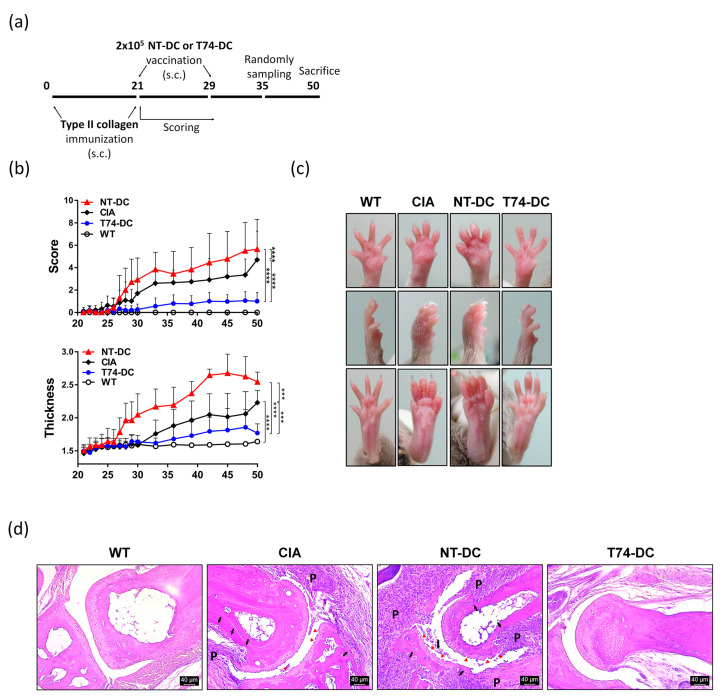
Type II collagen-primed T74-DCs attenuate CIA progression. DBA/1J mice were injected subcutaneously with 100 μL type II collagen (CII) emulsion. Mice were immunized boost dose with the same amount of collagen on Day 21 after the primary immunization. Established CIA mice were vaccinated with 2 × 10^5^ CII-primed T74-DCs, NT-DCs, or PBS twice on Days 21 and 29. (**a**) Schematic diagram of the animal experiment. (**b**) Arthritis incidence was observed by clinical scoring and footpad thickness from Day 21 until the end of experiment. Severity was scored on a scale of 0–4 for each paw. Data are expressed as the mean ± SEM (wild type: 6 mice, other groups: 10 mice). (**c**) Paws of mice from each group were randomly selected and photographed. Images show the front (**top**) and side (**middle**) of the front paws and the hind paws (**bottom**). (**d**) Paws removed from half mice in each group were fixed in 4% paraformaldehyde, decalcified for 3 weeks in HCl solution, and then embedded in paraffin. 5 μm thick serial sections were stained with H&E. Data are representative of the staining per each group (wild type: n = 3, other groups: n = 5). Bone and cartilage erosion are marked with black arrows and red arrowheads, respectively. P: pannus, I: inflammatory exudate. *** *p* < 0.001, **** *p* < 0.0001.

To evaluate the potential therapeutic effects of T74-DCs, CIA was induced in 8-week-old female DBA/1J mice. CII emulsified in adjuvant was immunized twice (subcutaneously, s.c.) into the mouse tail base (the second injection was given as a booster shot on Day 21 after the initial collagen injection). Established CIA mice were vaccinated twice (s.c.) on Days 21 and 29 with CII-primed T74-DCs or NT-DCs (Figure 5a). CIA control mice were injected with PBS, not DCs. After the second immunization, arthritis severity in the paws was scored. Interestingly, severity of arthritis in mice treated with CII-primed T74-DCs was significantly less than that in CIA control mice (Figure 5b). However, injection of NT-DCs accelerated arthritis symptoms. Paw thickness followed a similar trend. Figure 5c shows swelling, inflammation, and joint deformation of the paws in each group of mice. The paws of CIA control mice were very swollen. In addition, the paws of NT-DC-treated mice showed severe edema, as well as deformation of the toe joints. By contrast, the paws of T74-DC-treated mice showed only mild swelling. On Day 35 after the primary immunization, half of the mice from each group were sacrificed, and the paws were examined histologically. Compared with the paws of normal mice, those of CIA control mice showed mixed inflammatory cell infiltration, synovial hyperplasia, and pannus formation in the synovial lesions (Figure 5d). In addition, cartilage and cortical bone erosion occurred due to pannus invasion. Bone destruction and inflammation in paws of mice treated with CII-primed NT-DCs were more severe than in paws of CIA controls. However, paws of mice treated with CII-primed T74-DCs showed a clear articular space, without pathological damage and only mild cell infiltration. The histopathological scoring showed the same context (Figure S3). These data suggest that antigen-primed T74-DCs prevent CIA development and have a therapeutic effect on the development of arthritis.

### 3.6. Effect of T74-DC Therapy on T Cell-Mediated Immunity in CIA Mice

**Figure 6:**
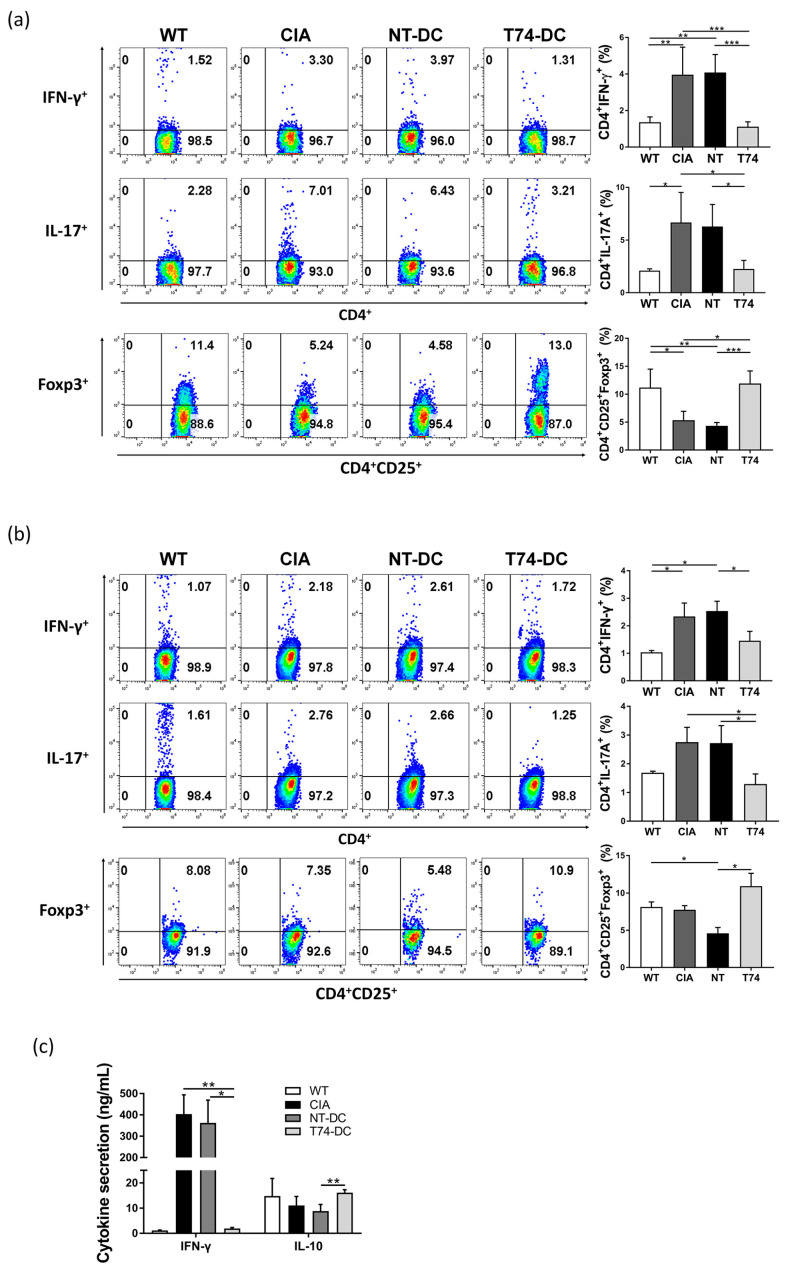
T74-DCs modulate systemic T cell-mediated immunity in CIA mice. (**a**) Splenocytes and (**b**) lymph node cells were isolated from each group of mice, cultured with CII (50 μg/mL) for 72 h, and analyzed by flow cytometry. The percentage of Th1, Th17, and Treg cells is shown on the dot plots. Data are representative of independent DC preparations. (**c**) Splenocytes from each group of mice were restimulated with CII and the culture supernatants were collected after 72 h. Th1- and Treg-related cytokine (IL-10 and IFN-γ) levels in culture supernatants were measured using commercial ELISA kit. Data in panels are expressed as the mean ± SEM (wild type: n = 3, other groups: n = 5). * *p* < 0.05, ** *p* < 0.01, *** *p* < 0.001. N/D: not detected.

To investigate immune status following injection of T74-DCs, we examined T cell subpopulations. Splenocytes and lymph node cells from each group of mice were restimulated for 72 h with CII (50 μg/mL) in RPMI containing 10% FBS. Harvested cells were stained and subpopulations were evaluated by flow cytometry. In CIA control mice, the Treg (CD4^+^CD25^+^Foxp3^+^) population in spleen was significantly lower than that in wild type (WT) mice (Figure 6a). The population in NT-DC-treated mice was similar to that in the CIA control. Interestingly, the number of splenic Tregs in T74-DC-treated mice recovered to WT levels. In addition, whereas the Th1/Th17 cell populations in mice injected with NT-DCs were higher than those in WT mice, they were reduced by T74-DCs. The lymph node cell subpopulations showed a trend similar to that observed for splenocytes (Figure 6b). Parallel assays revealed that Th1-related IFN-γ levels were, as expected, higher in CIA controls and NT-DC-treated mice than in WT mice. T74-DC therapy reduced production of IFN-γ (Figure 6c). Conversely, levels of anti-inflammatory IL-10 were higher in T74-DC-treated mice. Taken together, these results suggest that T74-DCs affect helper T cell differentiation, particularly in vivo Treg differentiation, and that T74-DCs ameliorate CIA development. These data support the in vitro data shown in Figure 2, and the histopathological findings.

## 4. Discussion

Rheumatoid arthritis (RA) is one of the most common autoimmune diseases. As TGF-β signaling plays a crucial role in immune tolerance and homeostasis, TGF-β receptor signaling agonists are a potential therapy for autoimmune diseases.

Until now, no chemically synthesized TGF-β receptor signaling agonists have been reported as a therapy for autoimmune disease. The development of a synthetic TGF-β receptor signaling agonist is meaningful from an economical perspective because chemical compounds can be mass-produced more easily than recombinant proteins.

We confirmed that T74 acts as a TGF-β receptor signaling agonist of DCs via phosphorylation of TGFBRI (Figure 1). T74-DCs expressed significantly lower levels of co-stimulatory molecules, MHC class II molecules, pro-inflammatory cytokines, and *Rsad2* and *Pdlim4* mRNA than NT-DCs (Figure 1 and Figure 3). Our previous studies revealed that *Rsad2* and *Pdlim4* are specific mDC markers [14,16]. Furthermore, T cell proliferation induced by T74-DCs was lower than that induced by NT-DCs (Figure 2d). These results imply that T74 blocks DC maturation and induces DC tolerance through interaction with TGFBRI.

T74 controls the immunostimulatory functions of DCs by inducing T cell polarization toward an immunosuppressive phenotype. Compared with NT-DCs, T74-DCs significantly reduced the Th1/Th17 populations but expanded the Treg population, both in vitro and in vivo (Figure 2 and Figure 6). T cell-related cytokines showed a parallel trend. In addition, in vivo experiments revealed that CIA mice treated with T74-DCs recovered systemic Treg populations to WT levels, which is consistent with the previous observation that Tregs are the main regulator of autoimmunity. It also confirms that defective Treg development is a main cause of inflammatory disease or autoimmunity [17]. Cell-penetrable mouse Foxp3 alleviates CIA in mice by converting CD4^+^CD25^+^ T cells into Treg-like cells and restoring the balance of Th17/Treg [18]. Another report suggests that ex vivo cultured antigen-specific Treg cells from CIA mice are stable in vivo, and that they reverse CIA progression by suppressing effector T cell proliferation [19]. Indeed, recent experiences of Treg cell therapy in patients show potential [20]. Based on these findings, we take the recovered Treg populations in our model as evidence that T74-DCs have a therapeutic effect.

The paws of CIA control mice showed inflammatory cell infiltration and invasive pannus formation, with bone and cartilage damage with synovial lesions. mDCs exposed to LPS but not T74 (NT-DCs) induced more severe pathological changes. By contrast, the joints of CIA mice injected with T74-DCs showed a clear articular space, similar to that in WT mice, demonstrating the antirheumatic effect of T74-DCs. The severity scores and paw thickness measurements support these findings (Figure 5). CIA attenuation by T74-DCs further strengthens the evidence that T74 induced DC tolerance.

We also confirmed whether T74 acts on the Smad pathway. Phosphorylation of Smad2 in T74-DCs was no different from that in NT-DCs, which is in contrast to the marked increase in Smad2 phosphorylation in rmTGF-β-treated DCs. However, T74 reduced phosphorylation of p38, JNK, ERK1/2, and NF-κB in DCs upon LPS stimulation (Figure 4). T74 suppresses phosphorylation of MAPKs and NF-κB to block DC maturation more strongly than activation of the canonical TGF-β/Smad signaling pathway. Non-Smad pathways are also reported to play a role in uncanonical TGF-β-mediated signaling, including the ERK, JNK/p38, RhoA, and PI3K/Akt pathways [21]. According to prior studies, non-Smad signaling by TGF-β is associated mainly with tumor progression rather than immunosuppression [21,22].

Here, for the first time, we show the tolerogenicity of T74-treated DCs as a therapeutic strategy for RA. Further studies should examine its applicability to other autoimmune animal models. The drugs that have been in the spotlight recently are TNF-α inhibitors or JAK inhibitors, which focus on alleviating inflammation caused by cytokine secretion. TGF-β signaling regulates fundamental aspects of the immune response by suppressing pathogenic immune cells. Our data suggest that T74 has similar capabilities. Thus, treatments based on T74-treated Tregs or tDCs can be developed.

## 5. Patents

1. Hyunjeong Kim, So Yeon Cho, Jimin Lee, Eosu Kim, Youngjoo Kwon, Soo-Yeon Hwang, Hyunji Jo, Seojeong Park, Younghwa Na. 2020. Composition for preventing, alleviating, or treating metabolic diseases. PCT/KR2020/003427, 12 March.
2. Youngjoo Kwon, Soo-Yeon Hwang, Hyunji Jo, Seojeong Park, Younghwa Na, Eosu Kim, Jihyeon Jeong, Minsun Park, Hyunjeong Kim. 2020. Composition for prevention, amelioration, or treatment of cancer. PCT/KR2020/006868, 27 May.

## Data Availability

The data supporting the findings of this study are available within the article and its supplementary materials.

## Supplementary Materials

The following supporting information can be downloaded at: https://www.mdpi.com/article/10.3390/cimb44090261/s1, Figure S1: Chemical structure of (E)-1-(3-Aminophenyl)-3-(2,5-dimethoxyphenyl)prop-2-en-1-one (T74), Figure S2: Cell viability and pro-inflammatory cytokine production by using T74 on DCs with various dosed, Figure S3: Histopathological inflammation scoring of H&E-stained tissue.
