# Supplementary material for: Synthetic TGF-β Signaling Agonist-Treated Dendritic Cells Induce Tolerogenicity and Antirheumatic Effects
Source: Curr Issues Mol Biol. 2022 Aug 24;44(9):3809–21. doi: 10.3390/cimb44090261 (PMC9498161; doi:10.3390/cimb44090261)
Supplement: Supplementary file 1 [file cimb-44-00261-s001.zip › cimb-1788487-supplementary.pdf]

Supplementary Materials

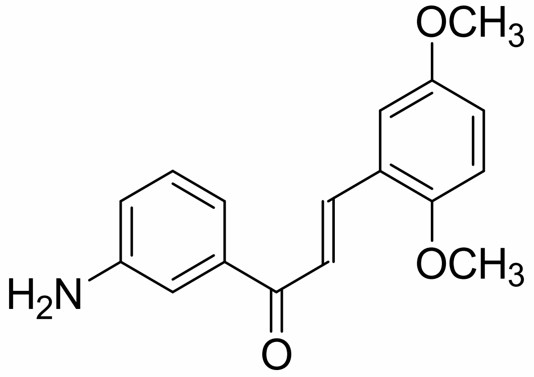

Figure S1. Chemical structure of (E)-1-(3-Aminophenyl)-3-(2,5-dimethoxyphenyl)prop-2-en-1-one (T74)

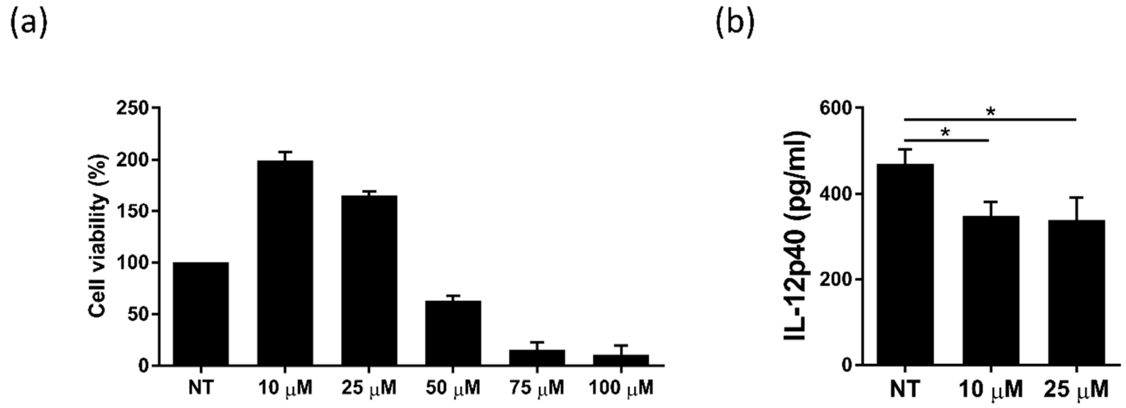

Figure S2. Cell viability and pro-inflammatory cytokine production by using T74 on DCs with various dosed. (a) DCs were treated with T74 at various doses from 10 μM to 100 μM. The cell viability was analyzed by WST-8 assay using EZ-Cytox (Dogen, Seoul, Korea) according to the manufacturer's instructions. (b) Pro-inflammatory cytokine secretion by T74-DCs was analyzed by ELISA. Data are expressed as the mean ± SEM. *$p < 0.05$.

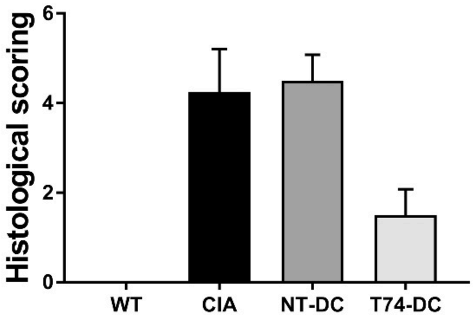

Figure S3. Histopathological inflammation scoring of H&E-stained tissue. The inflammation response was scored as follows: 0= normal; 1= minimal infiltration of inflammatory cells; 2= mild infiltration at 1 to 3 affected joint; 3= moderate infiltration with moderate edema; 4= marked infiltration affecting most areas; and 5= severe diffuse infiltration
